# Gas Nuisances and Their Removal

**Published:** 1857-07

**Authors:** 


### MISCELLANEA.

### GAS NUISANCES AND THEIR REMOVAL.

In a report on nuisances arising from gas-works, the Trades Committee of the Medical Officers of Health give several valuable remedial suggestions, herewith condensed.

Various sources of annoyance to a neighbourhood may arise from the manufacture of gas. 1. *Smoke.* The amount of smoke from the furnaces is usually insignificant, but that which proceeds from the openings at the roof of the retort-house is a nuisance, and is at its height when the retorts are being charged, or the coke withdrawn. The evolution of smoke is less during the charging of the single than of the continuous retorts. The former are charged in a few seconds, before the coals are sufficiently heated to part with much volatile matter. With the continuous retorts, there is a delay from the double charging at each end. Much of this might be avoided if two scoops at each end were used, both being ready filled before charging, the mouth-pieces being immediately and simultaneously adapted. The coke should be removed quickly, and at once quenched with water. Under the best arrangements a great deal of smoke is given off, which, with the steam, should be prevented becoming a nuisance. A remedy might be found in the closure of the roof openings, and in forming communications, at the upper part of the retort-house, with the chimney which carries off the heated air and smoke from the furnaces. 2. *The use of refuse lime from the purifiers* for luting the mouth-pieces is a source of nuisance, as it contains sulphur and other noxious compounds, and gives off, when heated, disagreeable vapours. This practice should be discontinued, and fresh lime, coke ash luting, or some other unobjectionable material, should be employed. 3. *Ammoniacal liquor and tar,* where the pits are uncovered, gives an offensive odour. The remedy is to cover the pits, and to convey these products without exposure to the barges. 4. *Revivification of the oxide of iron* causes an evolution of ammonia, favoured by a rise in temperature which the oxide undergoes during the process. This nuisance is less where effectual means of removing ammonia are employed prior to the passage of the gas to the oxide purifiers. Precautionary measures consist in the revivification of the oxide at short intervals, in the interception of the vapour that is given off, as at Equitable Works (Pimlico), and at the Chartered (Horseferry Road), and partly in the careful removal of ammonia from the gas by sulphate of lime, muriate of manganese, etc., prior to its being submitted to the action of the oxide of iron. Mr. Evans, of the Chartered Gas-works, however, thinks that ammonia, in moderate quantity in gas, is of advantage in neutralising the sulphurous acid formed during the combustion of the bisulphuret of carbon which gas always contains; and the presence of ammonia in gas is held, by some gas-engineers, to prevent the deposit of naphthaline in the small supply pipes. 5. *Refuse of the wet lime purifiers.* When the cream of lime from the purifiers is stored in pits, or in open tanks, the effluvia are most offensive. The carbonic acid of the atmosphere, also, acting upon the matter, increases the evolution of sulphuretted hydrogen and ammonia. A partial remedy for this nuisance is the use of water-tight covered tanks. 6. *Refuse from the dry lime purifiers.* The effluvia from a dry lime purifier, when opened, where no means of separating ammonia, sulphuretted hydrogen, etc., are previously employed, are overpoweringly offensive. Opening and emptying the purifier thus contaminates the atmosphere to a great distance. The dry lime purification should never be allowed unless effectual means of previously removing these compounds exist. When charged with offensive products it should be conveyed from the works in covered receptacles. 7. *The employment of the crude gas-tar for coating the gas-holders and iron work* is, in some instances, a source of offensive odour. An attempt should be made to discover some preservative which shall not possess similar disadvantages.

